# Resting state alpha oscillatory activity is a valid and reliable marker of schizotypy

**DOI:** 10.1038/s41598-021-89690-7

**Published:** 2021-05-17

**Authors:** Jelena Trajkovic, Francesco Di Gregorio, Francesca Ferri, Chiara Marzi, Stefano Diciotti, Vincenzo Romei

**Affiliations:** 1 Centro Studi e Ricerche in Neuroscienze Cognitive, Dipartimento di Psicologia, Alma Mater Studiorum – Università di Bologna, Campus di Cesena, 47521 Cesena, Italy; 2 UO Medicina Riabilitativa e Neuroriabilitazione, Azienda Unità Sanitaria Locale, 40139 Bologna, Italy; 3 Department of Neuroscience, Imaging and Clinical Sciences, “G. d’Annunzio” University of Chieti-Pescara, Chieti, Italy; 4 Institute for Advanced Biomedical Technologies, “G. d’Annunzio” University of Chieti-Pescara, Chieti, Italy; 5 Department of Electrical, Electronic, and Information Engineering “Guglielmo Marconi”, University of Bologna, Cesena, Italy; 6 Alma Mater Research Institute for Human-Centered Artificial Intelligence, University of Bologna, Bologna, Italy; 7 IRCCS Fondazione Santa Lucia, 00179 Rome, Italy

**Keywords:** Neuroscience, Cognitive neuroscience, Consciousness

## Abstract

Schizophrenia is among the most debilitating neuropsychiatric disorders. However, clear neurophysiological markers that would identify at-risk individuals represent still an unknown. The aim of this study was to investigate possible alterations in the resting alpha oscillatory activity in normal population high on schizotypy trait, a physiological condition known to be severely altered in patients with schizophrenia. Direct comparison of resting-state EEG oscillatory activity between Low and High Schizotypy Group (LSG and HSG) has revealed a clear right hemisphere alteration in alpha activity of the HSG. Specifically, HSG shows a significant slowing down of right hemisphere posterior alpha frequency and an altered distribution of its amplitude, with a tendency towards a reduction in the right hemisphere in comparison to LSG. Furthermore, altered and reduced connectivity in the right fronto-parietal network within the alpha range was found in the HSG. Crucially, a trained pattern classifier based on these indices of alpha activity was able to successfully differentiate HSG from LSG on tested participants further confirming the specific importance of right hemispheric alpha activity and intrahemispheric functional connectivity. By combining alpha activity and connectivity measures with a machine learning predictive model optimized in a nested stratified cross-validation loop, current research offers a promising clinical tool able to identify individuals at-risk of developing psychosis (i.e., high schizotypy individuals).

## Introduction

Schizophrenia is a highly debilitating and complex mental disorder characterized by impairments in integrating sensory and cognitive functions leading to incoherent perception. Schizophrenia often arises in late adolescence or early adulthood and is typically preceded by a high-risk (prodromal) phase, during which subtle neurocognitive impairments and sub-threshold psychotic symptoms usually emerge^1^. For this reason, increasing research efforts are focused upon identifying predictive neurobiological markers for early diagnosis in individuals at risk. Heightened risk for the development of a psychotic disorder is associated with schizotypal traits^2^. Substantial overlapping has been found between schizotypy and schizophrenia at genetic, biological and neurocognitive levels^3–5^, strongly supporting the claim of a continuous nature of schizotypy. Specifically, genome-wide association (GWA) research indicates that a vast number of independent polymorphisms confer risk^6–8^ for psychosis proneness, whereas schizophrenia represents the extreme of these multiple quantitative dimensions. These genetic factors explain about 50% of the schizotypic variance^3^, whereas the remaining variance can be explained by biological^9–12^ and psychosocial^13–16^ factors. Taken together, these common genetic and environmental underpinnings have led to the assumption that schizotypy reflects the subclinical expression of the symptoms of schizophrenia in the general population^17–19^.

Several studies of patients at different stages of schizophrenia, including the prodromal phases preceding the onset of the disorder, have recently reported abnormal spontaneous alpha oscillations and altered resting-state functional connectivity of the alpha rhythm^20–23^. Crucially, alpha rhythm is generated by a complex interplay between thalamic and cortical pacemakers and propagates via short and long range cortico-cortical, cortico-thalamic, and thalamo-cortical connections^24,25^. It is well known that large bursts of alpha (7–13 Hz) band activity dominate the human electroencephalogram (EEG) during periods of rest^26^. However, whether abnormalities of resting-state alpha rhythm are already present in individuals with high schizotypal personality traits, and can be taken as early risk predictors for these individuals, is still unknown. There is plenty of evidence in support of this hypothesis.

Murphy and Ongur^27^ reported decreased peak alpha frequency in first episode psychosis patients. Specifically, they found alpha slowing in posterior regions, while peak alpha frequency did not decrease significantly in frontal and temporal regions. Further supporting the clinical relevance of abnormal peak alpha frequency to schizophrenia, there is evidence available for therapeutic effects of individualized alpha frequency transcranial magnetic stimulation on the negative symptoms of schizophrenia^28^.

Moreover, according to the idea that schizophrenia originates as a disconnection syndrome^29^, first episode psychosis patients show abnormal functional connectivity, as estimated using the phase lag index (PLI), especially in the alpha rhythm. Hence, also alpha PLI, in addition to peak alpha frequency, seems to be valuable for producing clinical significance already at the onset of schizophrenia^20^.

Interestingly, alpha waves propagate from anterior-to-posterior and from the cortex to the thalamus^30–33^, so that cortico-cortical and cortico-thalamo-cortical connections allow frontal regions to drive posterior alpha activity. All in all, this evidence led to the idea that alpha rhythm plays an important role during top-down processing in healthy conditions^34–36^. Rest EEG connectivity studies specifically testing the association between abnormal directionality of the anterior-to-posterior propagating alpha rhythm and schizophrenia risk are currently unavailable though. However, the altered alpha band activity recorded in ultra-high-risk individuals during an auditory oddball task has been proposed to indicate that a deficit in top-down control exists before the onset of schizophrenia^37^. In sum, while there is now enough evidence available indicating that abnormal rest EEG alpha rhythm characterizes already the onset of schizophrenia, its potential role as an early marker of a predisposition toward schizophrenia in non-clinical populations is still poorly investigated^20,38^.

To fill this gap in the literature, in the present study we first established the association between sub-clinical schizotypy and specific indices of rest EEG alpha oscillatory activity (i.e., individual alpha peak frequency, IAF) and connectivity using both non-directional (i.e., weighted phase lag index, wPLI) and directional (i.e., time lag index, TLI) indices. The choice of investigating resting-state EEG features, rather than task-based EEG signals, has been motivated by both theoretical and practical reasons. From a theoretical standpoint, schizotypy is defined as a stable personality trait, thus possible alterations of EEG activity should be present already during resting-state. On a practical note, due to its simplicity and versatility, resting-state EEG recording can be considered an efficient screening tool that enables task-independent standardized measures for large scale assessments. Moreover, diagnostic accuracy in early onset psychosis and schizophrenia might be improved using machine learning approaches^39^, as already demonstrated in studies using genetic and neuroimaging features^6,40^. Similarly, machine learning methods allow to classify EEG features and thus identify clinical conditions based on EEG patterns. Indeed, recent machine learning studies in schizophrenia patients identified altered amplitudes and time lag in frontal event-related potentials^41^, lower levels of frontal alpha amplitude during working memory tasks^42^, functional alterations of alpha power spectrum over occipital-parietal and frontal areas^43^ and altered thalamo-cortical connectivity^44^. Therefore, we trained and tested a pattern classifier to create a predictive model able to assess the presence of high schizotypal traits based on the alpha resting state activity of an individual.

## Results

Resting-state EEG activity was recorded in a sample of 48 participants. Participants were divided into two groups based on the presence of schizotypal traits, estimated via Schizotypal Personality Questionnaire (SPQ)^45^. Two groups of 24 participants were subsequently created, based on SPQ score: a Low Schizotypal Group (LSG) with scores below the 20th percentile (Mean score: M = 7.62, Standard Error of the mean: SE = 0.52), and a High Schizotypal Group (HSG) with scores above the 80th percentile (M = 43.29, SE = 1.29). EEG was recorded from 64 scalp electrodes at rest for two minutes, while participants kept their eyes closed. An Independent Component Analysis (ICA) was performed for each participant to identify topographies reflecting activity in frontal and parieto-occipital areas for both the left and right hemisphere, representing our regions of interest (ROIs) for functional connectivity and alpha activity analyses.

### EEG features

**Figure 1:**
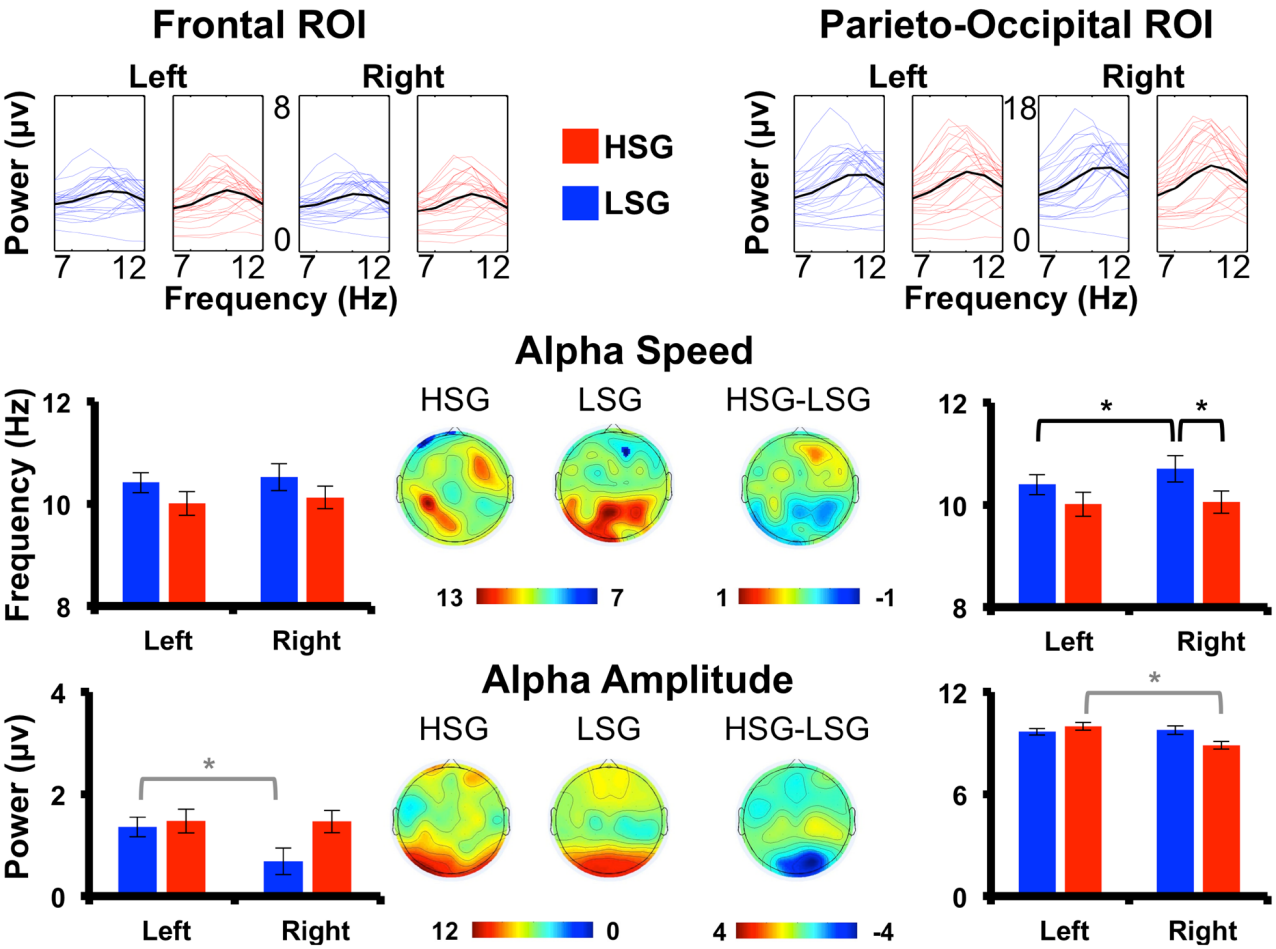
Individual Alpha Frequency (IAF) and Alpha Amplitude. First row: power spectra in the alpha frequency range (7–13 Hz) for left and right frontal and parieto-occipital regions of interest (ROI) divided for high schizotypy group (HSG) in red and low schizotypy group (LSG) in blue. Thin lines indicate single subject spectrum and black lines reflect group means. IAF. Observed alpha frequency peak in Hertz (Hz) for the two groups and for ROIs in both hemispheres (Left vs. Right). Topographies show scalp distribution of alpha frequency peak for the two groups and for the difference between groups. Alpha Amplitude. Observed maximum alpha amplitude in power (10*log10(μv)2) for the two groups and for ROIs in both hemispheres. Topographies show scalp distribution of alpha amplitude for the two groups and for the difference between groups. Corrected significant differences are marked with black asterisks. Uncorrected differences are marked with light-grey asterisks. Error bars represents Standard error of the mean. μv (microvolt); Hz (Hertz).

Power spectra over the ROIs, reflecting individual alpha frequency and amplitude (i.e., alpha activity) are shown in Fig. 1. In the following, t-test have been adjusted for multiple comparisons with corrected significance threshold *p* value of 0.013 for four comparisons (for details see methods). Analyses show a general faster alpha frequency, across groups (HSG vs. LSG) and ROIs (frontal and parieto-occipital), in the right hemisphere (M = 10.38 Hz, SE = 0.12 Hz) compared to the left hemisphere (M = 10.25 Hz, SE = 0.11 Hz) (main effect of hemisphere *F*(1,46) = 10.37, *p* = 0.002, *pη*^2^ = 0.18, 90% CI [0.03; 0.37]). More in detail, a significant three-way interaction (Group x Hemisphere x ROIs, *F*(1,46) = 4.44, *p* = 0.040, *pη*^2^ = 0.09, 90% CI [0.01; 0.23]) suggests that this hemispheric asymmetry in alpha frequency is more prominent at a parieto-occipital relative to frontal level, and specific for the LSG (M_left_ = 10.39 Hz, SE = 0.14 Hz; M_right_ = 10.70 Hz; SE = 0.17 Hz) (one-tailed *t*(23) = 2.48, *p* = 0.011, Effect size Cohen’s *d* = 0.51, 90% CI [0.14; 0.86]). Interestingly, at a parieto-occipital level also a group difference emerged, with faster alpha frequency in LSG (M = 10.70 Hz, SE = 0.17 Hz) compared to HSG (M = 10.09 Hz, SE = 0.17 Hz) (one-tailed *t*(46) = 2.60, *p* = 0.006, *d* = 0.75, 90% CI [0.25; 1.24]).

In line with the notion of a postero-anterior gradient^46^, our analyses also showed greater alpha amplitude over the parieto-occipital ROI (M = 9.50μv, SE = 0.95μv) compared to the frontal ROI (M = 1.26μv, SE = 0.94μv) (main effect of the ROI, *F*(1,46) = 135.43, *p* < 0.001, *pη*^2^ = 0.75, 90% CI [0.63; 0.81]). Additionally, as for alpha frequency, a similar hemispheric asymmetry emerged for alpha amplitude (main effect of hemisphere *F*(1,46) = 5.13, *p* = 0.028, *pη*^2^ = 0.10, 90% CI [0.03; 0.32]). In particular, the right hemisphere shows lower alpha amplitude (M = 5.16μv, SE = 0.88μv) compared to the left hemisphere (M = 5.60μv, SE = 0.88μv). Importantly, the mentioned patterns of alpha amplitude are differently modulated between groups (significant three-way interaction *F*(1,46) = 6.30, *p* = 0.016, *pη*^2^ = 0.12, 90% CI [0.01; 0.27]). Specifically, the described inter-hemispheric asymmetries in the alpha amplitude distribution are present in the LSG only over the frontal ROIs, with a higher alpha amplitude in the left hemisphere (M = 2.03μv, SE = 1.27μv) compared to the right hemisphere (M = 1.13μv, SE = 1.22μv) (*t*(23) = 2.51, *p* = 0.019, *d* = 0.51, 90% CI [0.15; 0.86]) which however, does not survive multiple comparisons (corrected significance threshold *p* = 0.013; see Methods). On the other hand, there is an analogous asymmetry shifted back over the parieto-occipital ROI in the HSG (M_left_ = 9.39μv, SE = 1.51μv; M_right_ = 8.37μv, SE = 1.58μv) (*t*(23) = 2.09, *p* = 0.048, d = 0.43, 90% CI [0.07; 0.77]), which however, does not survive multiple comparisons (corrected significance threshold *p* = 0.013; see Methods).

**Figure 2:**
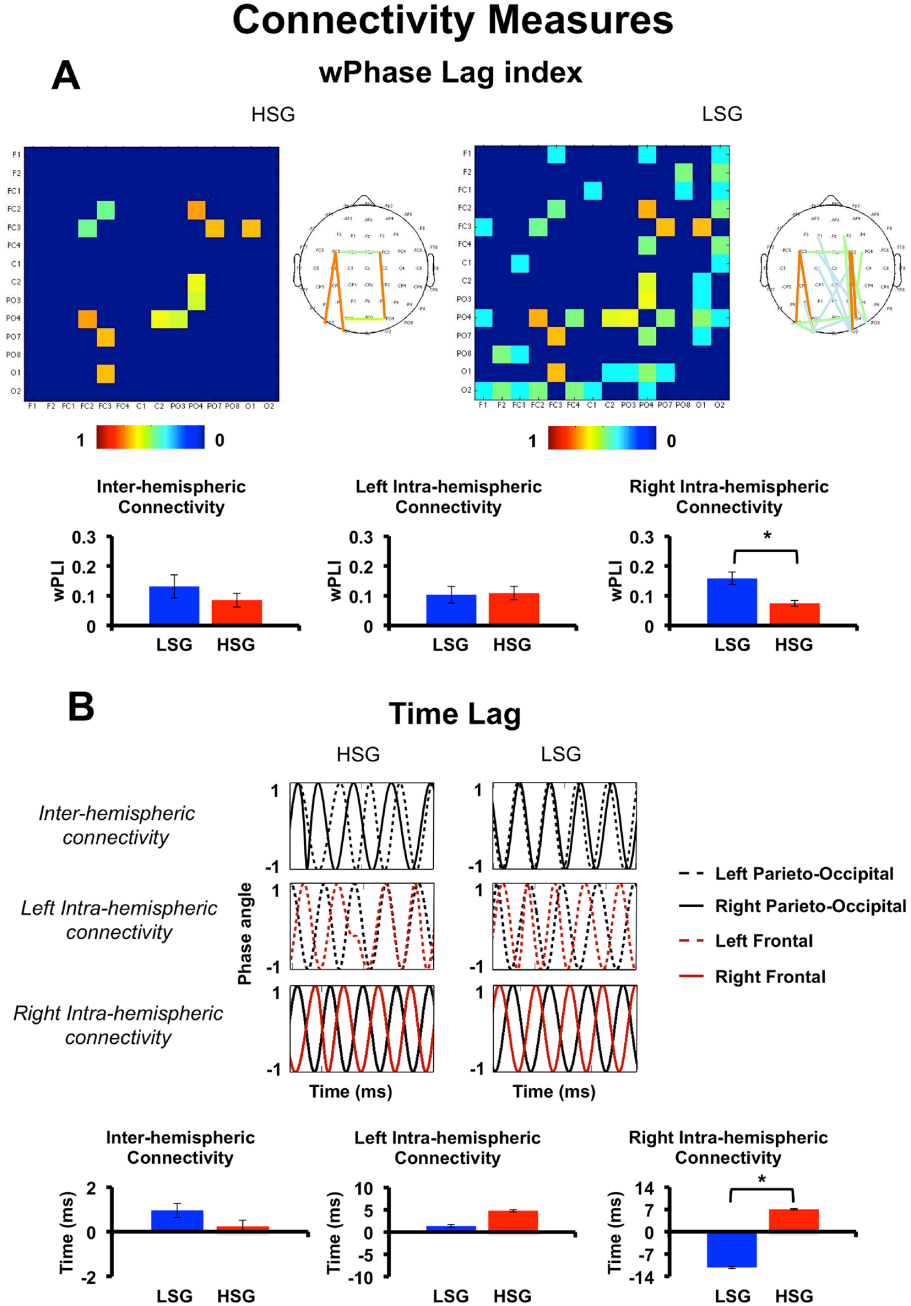
Connectivity Measures. (**A**) Correlation matrices of the weighted Phase Lag Index for the High Schizotypy (HSG) vs. Low Schizotypy (LSG) Groups in the selected subclusters of electrodes in the frontal and parieto-occipital ROIs. Topographies show grand mean wPLI connectomes for each electrode with wPLI > 0 (non-random connectivity). Bar graphs below show the corresponding mean wPLI in the selected ROIs for connectivity analyses (see methods). (**B**) Phase angles distributions over time, plotted as sinusoid oscillations, in the selected ROIs for connectivity analyses based on time lag. First row shows oscillations in left and right parieto-occipital ROIs (inter-hemispheric connectivity) for HSG and LSG. Second and third rows show oscillations in the frontal and occipito-parietal ROIs in the left and right hemisphere respectively (intra-hemipsheric connectivity). Bar graphs below show the corresponding mean Time lags for left and right inter-hemispheric and intra-hemispheric connectivities in both groups. Error bars represents Standard error of the mean. ms (milliseconds); Hz (Hertz).

Functional connectivity across groups (LSG and HSG) was estimated based on phase connectivity measures between ROIs in the alpha frequency peak (Fig. 2). In the following, t-test have been adjusted for multiple comparisons with corrected significance threshold *p* value of 0.017 for three comparisons (for details see methods). An inter-group difference in the wPLI was found over the fronto-parieto-occipital connectivity in the right hemisphere (*t*(46) = 3.26, *p* = 0.002, *d* = 0.94, 90% CI [0.43; 1.44]), with the HSG showing a lower value of wPLI compared to the LSG (M_high_ = 0.08, SE_high_ = 0.01; M_low_ = 0.15, SE_low_ = 0.02). No differences in connectivity were found between groups over the other considered ROIs (all *ts* < 0.90, all *ps* > 0.37, all *ds* < 0.26). Analysis of the time lag yielded similar results, further confirming the nature of the differential effect between HSG and LSG selectively observed between frontal and parieto-occipital ROIs in the right hemisphere (*t*(46) = 2.85, *p* = 0.006, *d* = 0.82, 90% CI [0.32; 1.31]). More in detail, the mean values of the TLI suggest that not only timing, but also the direction of communication in the right hemisphere is significantly different in the two groups (M_high_ = 7.04 ms, SE_high_ = 4.67 ms; M_low_ = − 11.21 ms, SE_low_ = 4.37 ms). Indeed, while in the LSG the phase of the frontal precedes the phase of the parieto-occipital ROI, in the HSG the opposite trend is observed.

Taken together, EEG data show group differences in the right hemisphere in terms of alpha speed (slower alpha in the HSG) and in the hemispheric distribution of alpha amplitude (asymmetry shifted from frontal to parieto-occipital ROIs for HSG compared to LSG). Moreover, connectivity measures in the HSG support the disconnection syndrome hypothesis^29^ by pointing to a reduced and even altered fronto-parieto-occipital connectivity in the right hemisphere.

### Machine learning pattern classifiers

**Figure 3:**
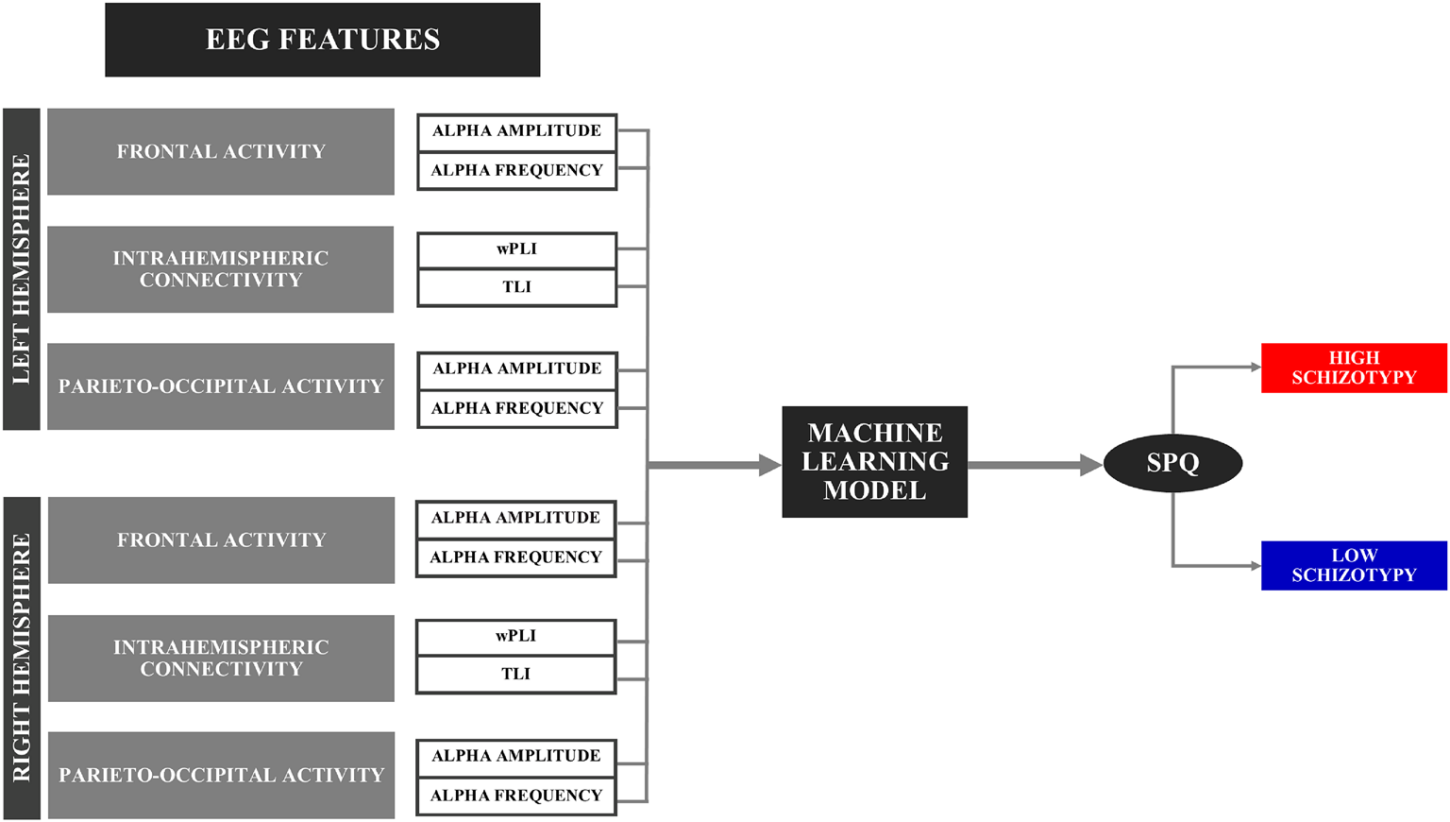
EEG features included in the machine learning pattern classifier.

According to information gathered from the literature on EEG markers of schizophrenia, a pattern classifier has been trained and tested with the aim of creating a predictive model able to assess the presence of schizotypal traits based on the alpha resting state activity and connectivity (Fig. 3). In particular, we have looked at alpha peak frequency, shown to be generally reduced in schizophrenia^21,27^, alpha amplitude, also shown to be altered in schizophrenia^23,47^, and measures of fronto-parietal connectivity (including wPLI and TLI), as schizophrenia has been defined as a functional disconnection syndrome within the default mode network encompassing fronto-parietal networks^20,48,49^.

**Figure 4:**
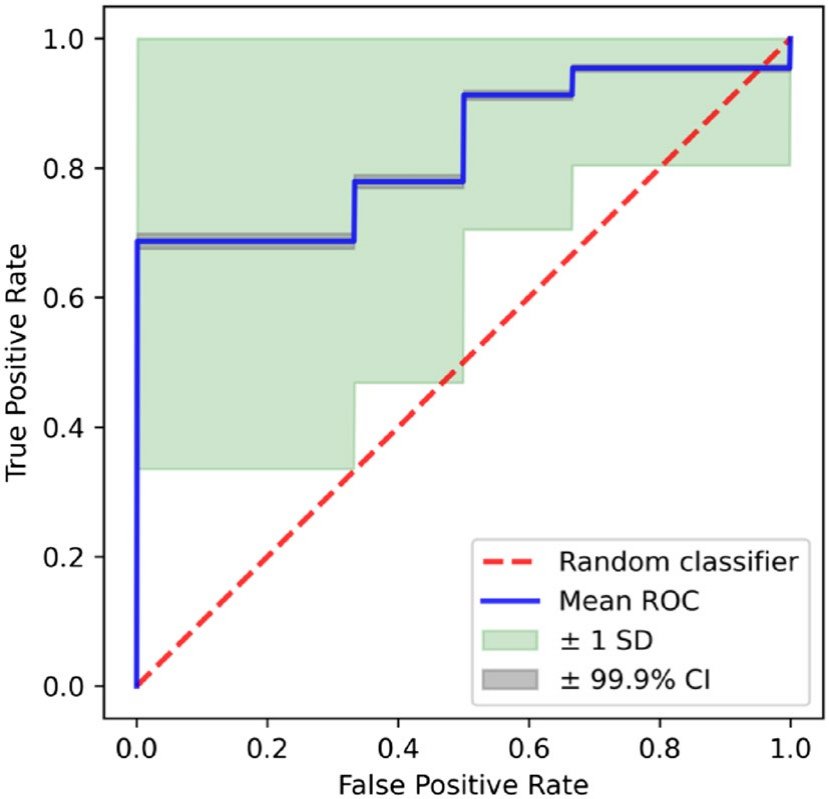
Average ROC curve. The average ROC curve across 10 000 outer loops of the nested CV (10 folds × 1000 repetitions) along with the standard deviation and the 99.9% confidence interval of the average is shown.

**Figure 5:**
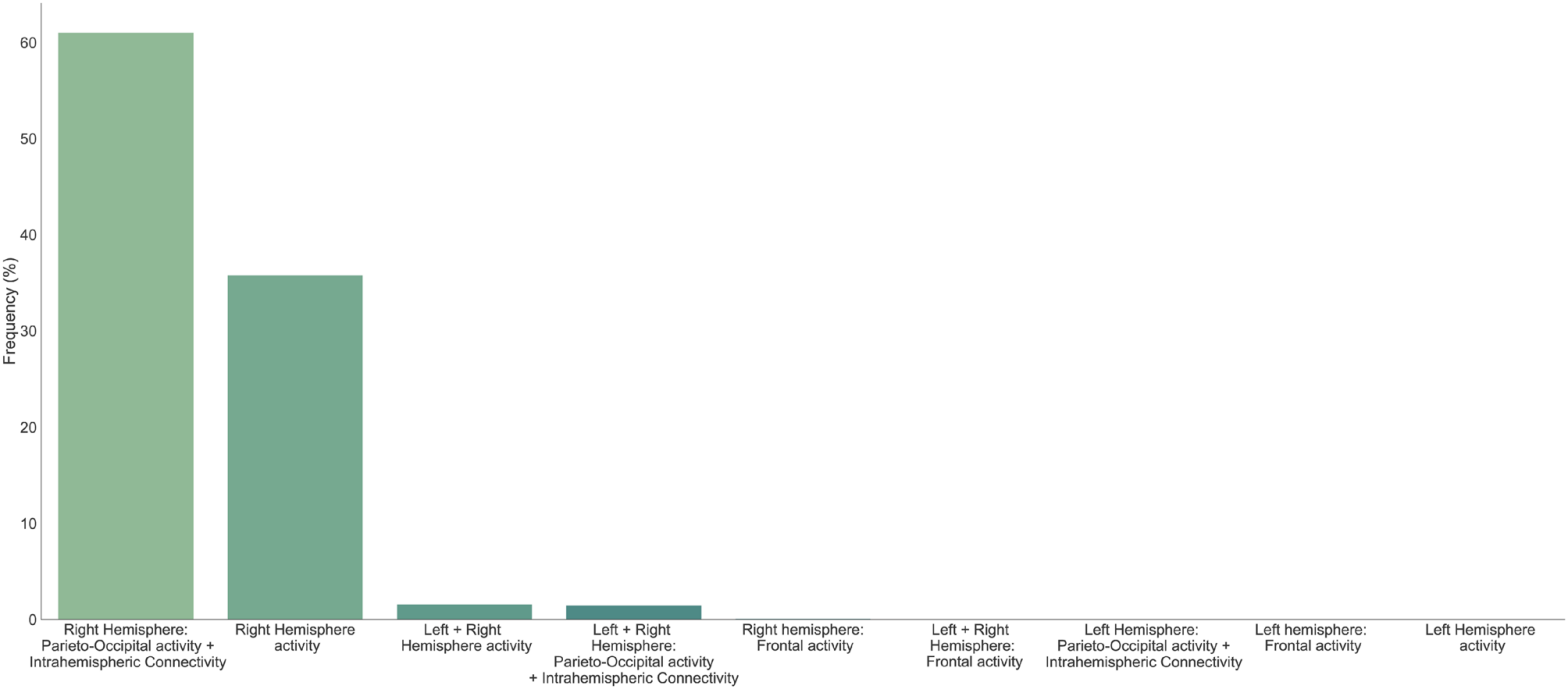
Ranking of feature selection. The relative frequency (%) with which each feature combination was selected across all outer CV folds in 1000 repetitions is shown. The feature combinations have been reordered on the occurring frequency.

Moreover, in order to distinguish between HSG and LSG, these features were included as possible input features of the models either for both right and left hemisphere or separately for each hemisphere. At the same time, we looked separately at frontal and posterior features with particular focus on posterior alpha activity and intrahemispheric connectivity. This was determined by the fact that literature on schizophrenia has shown an important discrepancy between studies supporting a general alteration of these features^22,23,50^, other pointing to a more specific hemispheric alteration^20,38,51,52^ and finally findings revealing altered alpha indices specifically at posterior sites and excluding frontal regions in patients with first episode psychosis^27^. Using a tenfold nested cross-validation (CV) procedure, repeated 1000 times, in the test sets of the outer CV, we observed a sensitivity of 78.8% (4.9%) [mean (standard deviation)], specificity of 69.7% (5.2%), balanced accuracy of 74.3% (3.8%) and area under the receiver operating characteristic – curve (AUC) of 0.83 (0.04). The plot of the average ROC curve across 10 000 outer loops of the nested CV (10 folds × 1000 repetitions) along with the standard deviation and the 99.9% confidence interval of the average is shown in Fig. 4. The ranking of the best feature combination has been reported in Fig. 5.

The first three combinations included only the right hemisphere activity and, overall, accounted for the 96.2% of the total occurrences. The remaining 3.8% was accounted for by the left + right hemisphere activity. Feature combinations including only left features were never selected.

In general, machine learning results suggest that differences between groups are maximal over the right hemisphere, or in other words, that right hemispheric features of alpha activity are the best predictors of schizotypal traits.

## Discussion

In the present study, we used a machine learning approach to identify biomarkers able to predict which individuals are at higher risks of developing schizophrenic symptoms. To the best of our knowledge, our study represents the first application of machine learning techniques to investigate resting-state EEG features in assessing the presence of high schizotypy traits. In particular, we investigated possible alterations of resting-state alpha oscillatory activity, known to be impaired in patients with schizophrenia, in the healthy population either high or low in schizotypy traits. To this aim, different parameters of resting-state alpha band activity have been included in the study: (1) alpha-amplitude (2) individual alpha frequency (IAF) (3) and indices of phase connectivity between distant neural ensembles within the alpha frequency range. Additionally, measures of alpha-amplitude and IAF have been investigated separately across the two hemispheres (left and right clusters of electrodes) and individually for two different regions of interest (frontal and parieto-occipital clusters), along with both interhemispheric (between parieto-occipital electrodes of left and right hemisphere) and intrahemispheric (between frontal and parieto-occipital electrodes of both left and right hemisphere) measures of alpha phase connectivity. Crucially, the above-mentioned indices of alpha activity were used as input features of a state-of-the-art pattern classifier, with the aim of building a model able to predict the presence of schizotypy traits of an individual based on resting-state alpha activity, thus even establishing a distinct and influential role of alpha oscillatory activity as electrophysiological marker of schizotypy dimension. The pattern classifier used a nested stratified CV loop to perform, at the same time, in the inner CV loop, selection of the best feature combination in discriminating the presence of schizotypal traits, as well as the best classifier (between C-SVM with linear kernel and logistic regression with L^2^ penalty) along with their hyper-parameter optimization.

Our results indicate that alterations of IAF are present in the high schizotypy group. In particular, high schizotypy seems to be accompanied by a decreased IAF in the right occipital component. The slowing of resting-state alpha activity has been reported in schizophrenia patients^21^, as well as in first episode psychosis^27^. Here, we found that this slowing is present also in the high schizotypy population, but restricted to the right parieto-occipital region.

In addition to these changes in IAF, high schizotypals also demonstrated an asymmetry of alpha-amplitude in parieto-occipital regions, with reduced alpha-amplitude in the right hemisphere. Diminished resting-state alpha-amplitude has previously been found both in individuals with psychotic disorders and their healthy relatives^22,23^, although even opposite results have been reported^47^, as well as null differences between first episode psychosis patients and healthy controls^27^. These discrepancies could be due to between-study differences in methodology and EEG data processing, illness chronicity or diagnostic heterogeneity, some of which could be clarified by systematically investigating the identified biomarkers both in clinical and subclinical populations.

Finally, measures of long-range connectivity in the alpha range have shown a distinct alteration in HSG. Specifically, similar to IAF results, differences between groups emerge only in the right hemisphere, with a reduced connectivity between frontal and parieto-occipital areas in HSG as measured by wPLI that, furthermore seems to follow an opposite parieto-frontal direction as shown through TLI measures. Moreover, no differences in interhemispheric connectivity have been observed between HSG and LSG. Altogether connectivity analyses point to reduced resting-state functional communication between frontal and parietal areas within the alpha range in the HSG, in line with previous results describing a similar pattern in schizophrenia patients^20^ but restricted to the right hemisphere in schizotypy.

In order to affirm that high schizotypals can be identified on a single subject level based on neural indices, thus by observing their resting-state alpha activity, a pattern classifier has been trained and tested which, if the hypothesis holds, should be able to successfully differentiate between HSG and LSG. Apart from being able to predict the participants group membership (low vs. high schizotypy) based on all the examined features of resting-state alpha activity (74.3% of balanced accuracy), two interesting dissociations have emerged. The first one concerns frontal and parieto-occipital features, with solely the latter being able to successfully differentiate between the two groups (frontal features do not seem to contribute to differentiating the two groups; see Fig. 5). This outcome is in line with previous findings which have not revealed altered alpha indices over frontal regions in patients with first episode psychosis^27^. Secondly, differences in alpha activity between the HSG and LSG seem to be present only in the right hemisphere (best feature rankings including only left features were never selected). Throughout the years, several theories have been proposed describing schizophrenia as an interhemispheric imbalance. Amongst the proposed genesis of this imbalance, both hypo-functioning and/or hyper-functioning of the one hemisphere have been hypothesized^53–55^, although often without firm empirical grounds to dissociate these alternatives. Our results support an altered pattern within the right hemisphere showing a slower and reduced alpha activity and an exclusively intrahemispheric altered communication between frontal and parietal regions. Following the dimensional approach, one could hypothesize either that the right hemisphere dysfunction could be more pronounced in schizophrenia, or alternatively, can instantiate the insurgence of first psychotic symptoms by spreading the right hemisphere dysfunction to the left hemisphere, thus resulting in a more generalized disconnection syndrome. Current research in schizophrenia does not point to the idea of a hindered right hemispheric activity but to a more spread dysconnectivity so future research should systematically point to identify whether the neurophysiological prodromal phase leading from a subclinical to a clinical psychotic condition may reside in an interhemispheric spreading.

## Conclusions

Overall, our results clearly demonstrate that the altered patterns of resting-state alpha activity observed in schizophrenia patients can be tracked already before the onset of the psychosis. Specifically, we observe the presence of an altered pattern concerning the resting-state alpha oscillatory activity in the high, relative to the low schizotypy population. Thus, alpha activity seems to represent an important electrophysiological marker, which may likely pave a higher risk of developing schizophrenia spectrum psychopathology according to specific indices as pointed out by our study. Interestingly, these differences are most evident in the right posterior region and its’ functional connections with the right anterior cortex. The right parieto-occipital deficit and fronto-parietal disconnection syndrome in the HSG may significantly alter both sensory processing per se, but also top-down influence on controlling sensory processing. Therefore, this research offers a firm ground to future investigations to identify patterns of neural and cognitive developments anticipating at high-risk individuals and in describing neurocognitive (dis)functioning across the schizophrenia spectrum.

Although representing a valid tool for detection and measurement of schizotypy^45^, SPQ still represents a self-report questionnaire, thus facing various methodological issues^56,57^. Crucially, current research has employed computational methods in order to affirm the relevance of identified oscillatory features as important electrophysiological markers of schizotypy. Specifically, by building a pattern classifier, we were not only able to describe the existence of possible differences in alpha activity between HSG and LSG, but also to demonstrate their ability to successfully identify individuals high in schizotypy. Thus, this approach offers a novel accurate diagnostic tool able to detect biomarkers defining at-risk individuals of developing schizophrenia spectrum disorders, based on resting state alpha activity. In addition, the inclusion of other features (e.g., genetic and neuroimaging data) would likely enhance the over-all performance of the model, although not always feasible, due to time and resource constraints. Moreover, we note that due to the relatively small sample, it would be interesting to confirm the results obtained with our built pattern classifier by extending future machine learning applications to an independent and wider sample of participants. The availability of larger datasets will also pave the way to the adoption of deep learning approaches which may improve the overall performance.

The main aim of the current study was to identify EEG configurational pattern of activity that could represent a fingerprint of schizophrenia proneness. As such, the focus of this study was not on comparing EEG activity across different mental disorders. Therefore, by using state-of-the-art machine learning analysis of the EEG patterns implemented here, future studies should empirically test whether the EEG activity pattern identified here is specific for schizophrenia risk or rather represents a transdiagnostic biomarker of risk for psychopathology.

Finally, the question remains how do altered patterns in alpha activity during rest translate into relevant cognitive processing? Both alpha amplitude and long-range fronto-parietal alpha synchronization have a well-determined roles in visuo-attentional inhibition and selection^58^, along with occipital alpha peak frequency acting as temporal and spatial sampling mechanism^59–66^. Therefore, should we expect these altered patterns to persist even during visuo-attentional tasks, leading to reduced attentional efficiency and altered perception in schizotypy? Future research is expected to address these questions, establishing a tight link between schizotypy and schizophrenia, thus enabling an accurate and detailed description of early markers of psychosis.

## Materials and methods

### Participants

Participants were selected on a sample of 350 students from the University of Bologna based on the presence of schizotypal traits, estimated via Schizotypal Personality Questionnaire (SPQ). Two age- (*t*(46) = 1.56, *p* = 0.124, *d* = 0.45, 90% CI [− 0.12; 1.02]) and gender-matched (*χ*^2^ = 0.76, *p* = 0.38) groups of 24 participants were subsequently created, based on SPQ score: a Low Schizotypal Group (LSG) with scores below the 20th percentile (Mean score: M = 7.62, Standard Error of the mean: SE = 0.52), and a High Schizotypal Group (HSG) with scores above the 80th percentile (M = 43.29, SE = 1.29) who agreed to take part in the present study. As a result, a sample of 48 participants (see Table 1 for detailed demographics) was recruited for electrophysiological data collection.

**Table 1:** Demographics.

	LSG	HSG
Participants	24	24
Mean age (yrs) (*SEM*)	23.12 (0.50)	22.08 (0.44)
Males	9	12
Females	15	12
SPQ score range (*M*)	3–11 (7.62)	32–57 (43.29)

Each group counted one left-handed subject. All participants signed a written informed consent prior to take part in the study, which was conducted in accordance with the Declaration of Helsinki^67^, and approved by the Bioethics Committee of the University of Bologna. All participants had no neurocognitive or psychiatric disorders.

### EEG recordings

Participants were comfortably seated in a room with dimmed lights. EEG was recorded at rest for two minutes, while participants kept their eyes closed^68^. A set of 64 Ag/AgCl electrodes was mounted according to the international 10–20 system (Fast’n Easy-Electrode, Easycap, Herrsching, Germany). Additionally, four EoG channels were positioned: on the outer canthi of both eyes, as well as above and below the left eye. The right and left mastoids were used as the online and off-line reference, respectively. Ground was positioned on the right cheek of the subject. All impedances were kept below 10 kΩ. EEG signals were recorded with a pass band filter 0.5–50 Hz (as set in Brain Vision Recorder, Brain Products, Gilching, Germany) at a sampling rate of 1000 Hz. Off-line data were resampled at 500 Hz (function pop_resample on EEGLab) and re-referenced to the average of all electrodes.

All EEG analyses were implemented by custom-made routines developed in Matlab R2013a (The MathWorks, Inc., Natick, Massachusetts, United States) using EEGLab toolbox functions (v. 13.0.1)^69^.

### EEG data processing

**Figure 6:**
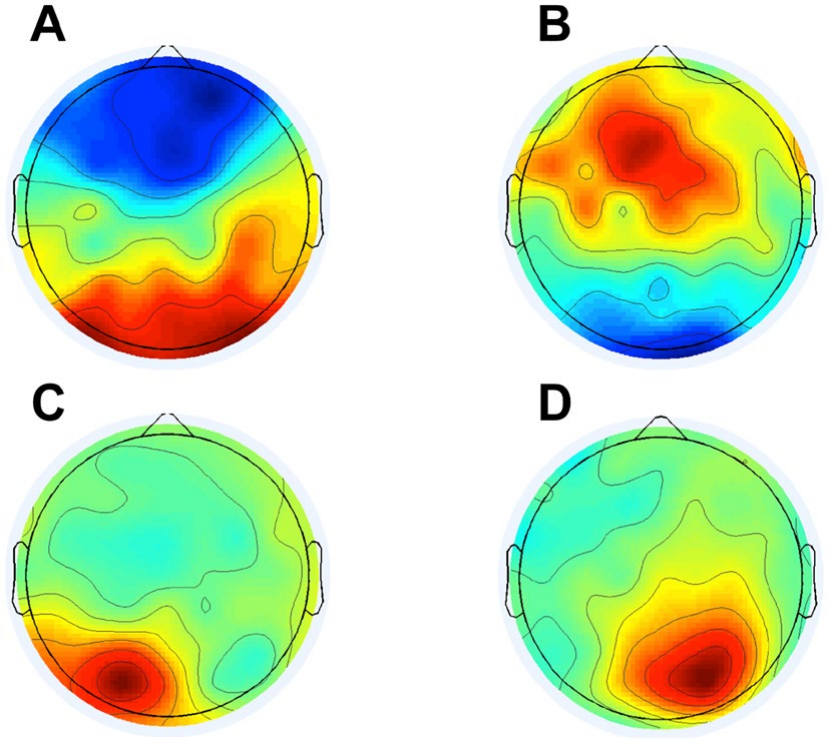
Regions of interest (ROIs). Templates of relevant components for frontal (**A**, **B**), left parieto-occipital (**C**) and right parieto-occipital (**D**) components.

Resting state EEG data were band-pass filtered (using a Hamming windowed sync FIR filter implemented in the pop_eegfiltnew function on EEGLab) for alpha frequency 6 to 14 Hz, and epoched in 2000 ms temporal windows. An Independent Component Analysis (ICA) was performed for each participant to identify topographies reflecting activity in frontal and parieto-occipital areas for both the left and right hemisphere, representing our regions of interest (ROIs) for alpha analysis (see Fig. 6). ICA method separates EEG data on distinct information sources (i.e., independent components) and provides the weighted projection from each independent component to each scalp electrode^70–72^. In particular, individual alpha frequency peak (IAF) and alpha amplitude were extracted from individual power spectra separately over each ROI in subclusters of electrodes selected by visual inspection of the identified topographies: frontal ROIs (left electrode cluster: F1, FC1, C1, FC3, right electrode cluster: F2, FC2, C2, FC4), as well as parieto-occipital ROIs f (left electrode cluster: PO7, PO3 and 01, right electrode cluster: PO8, PO4 and O2). Weighted phase lag Index (wPLI)^73^ and time lag were used as indices of connectivity. In particular, inter-hemispheric connectivity was estimated between right and left parieto-occipital ROI and intra-hemispheric connectivity was estimated in both hemispheres between frontal and parieto-occipital ROIs.

First, four templates, reflecting spatial topography of the ROIs, were identified via visual inspection within all ICs: two central frontal components and two parieto-occipital components, one for each hemisphere (left and right). Subsequently, for each subject, relevant ICs were identified via automatic multistep correlational template matching (CORRMAP, 0.80 correlation threshold)^74^. Topographies of ICs labeled as frontal and parieto-occipital components were visually inspected and back-projected to the data for frequency, amplitude and connectivity analyses^75^. For each participant, a minimum of one and maximum of three components were identified per template.

### EEG features extraction

Individual alpha peak and alpha amplitude were extracted from power spectra of each participant using an automated peak-detection algorithm (function RestingIAF on EEGLab)^76^. This algorithm uses a Sovitzky-Golay filter (SGF, frequency resolution 0.24 Hz, polynomial order 5 of the SGF), which smooth power spectra and attenuate random noise. Alpha amplitude was defined as the maximum alpha power, expressed in normalized power (10*log_10_(μv)^2^). To calculate wPLI, EEG resting state data were divided into 2500 ms non-overlapping windows^77^. Then the cross-spectrum of the time series signals was calculated and the wPLI estimates the magnitude of the imaginary part of the cross-spectrum. For each participant, wPLI was estimated as a function of individual alpha frequency peak and 14X14 connectivity matrices were generated over the selected electrode clusters (see above). Time lag estimates the mean difference in milliseconds of two time series spectra.

### EEG features

Individual independent components (ICs) were analyzed in order to extract electrophysiological features both for frontal and occipito-parietal regions of interest (ROIs) in the right and left hemisphere to be entered in the machine learning pattern classifier.

The following EEG features were extracted:

### Individual alpha frequency (IAF)

For each participant, IAF was defined as the exact frequency in the alpha range (7–13 Hz) containing the maximum power. It was extracted from the individual power spectra in the alpha range and calculated using an automated peak-detection algorithm (function *RestingIAF* on EEGLab)^76^.

### Alpha amplitude

For each participant, alpha amplitude was defined as the maximum power in the alpha range (7–13 Hz), expressed in normalized power (10*log_10_(μv)^2^).

### Weighted phase lag index (wPLI)

This feature was extracted to calculate functional connectivity in the alpha range. This is a measure of phase-based connectivity calculated in a specific frequency, which accounts only for non-zero phase lag/lead relations between two time series signals^73^. wPLI is calculated between two neurophysiologic signals and can assume values between 0 and 1. Larger values of wPLI reflect a consistent phase relation between two signals. If the relation between two signals is random, the wPLI value is 0. Connectivity between frontal and parieto-occipital ROIs in the right hemisphere was estimated on the averaged wPLI values calculated over the following electrode clusters: right frontal ROI (F2,FC2,C2,FC4) and right parieto-occipital ROI (PO8,PO4,O2). Similarly, left fronto parieto-occipital connectivity was estimated over left frontal ROI (F1,FC1,C1,FC3) and parieto-occipital ROI (PO7,PO3,O1).

### Time lag index (TLI)

This feature adds a further dimension to the wPLI as it provides information about the directionality of the communication between two synchronized signals^78^. It represents the means of the temporal phase lag in the cross-spectrum between time series signals of the selected clusters and, unlike the wPLI, it offers further insight regarding the temporal dimension of the synchronization. Specifically, TLI is used to determine the averaged phase differences in milliseconds of two considered signals^78^. Positive values of the TLI indicate a lag in the phase of the first considered signal with respect to the other, thus indicating the directionality of the communication between two synchronized signals.

### EEG data analysis

2 × 2 × 2 mixed-model ANOVAs were performed on IAF and alpha amplitude, with the between subject factor GROUP (HSG, LSG) and the within subject factors HEMISPHERE (left and right) and ROI (frontal and parieto-occipital ICs). Specific differences in the alpha activity were further tested both for alpha frequency (with paired and independent samples one-tailed t-tests as a directionality hypothesis was formulated^14^) and alpha amplitude (with paired and independent samples two-tailed t-tests). Between groups planned comparison were performed on wPLI and TLI using independent samples two-tailed t-tests. *p* values < 0.05 were considered significant, along with Dunn-Sidak correction procedures for multiple comparison being applied where necessary^79^, with a corrected significance threshold *p* value of 0.013 for four comparisons (alpha activity analyses) and a corrected significance threshold *p* value of 0.017 for three comparisons (connectivity analyses).

### Machine learning pattern classifiers

**Figure 7:**
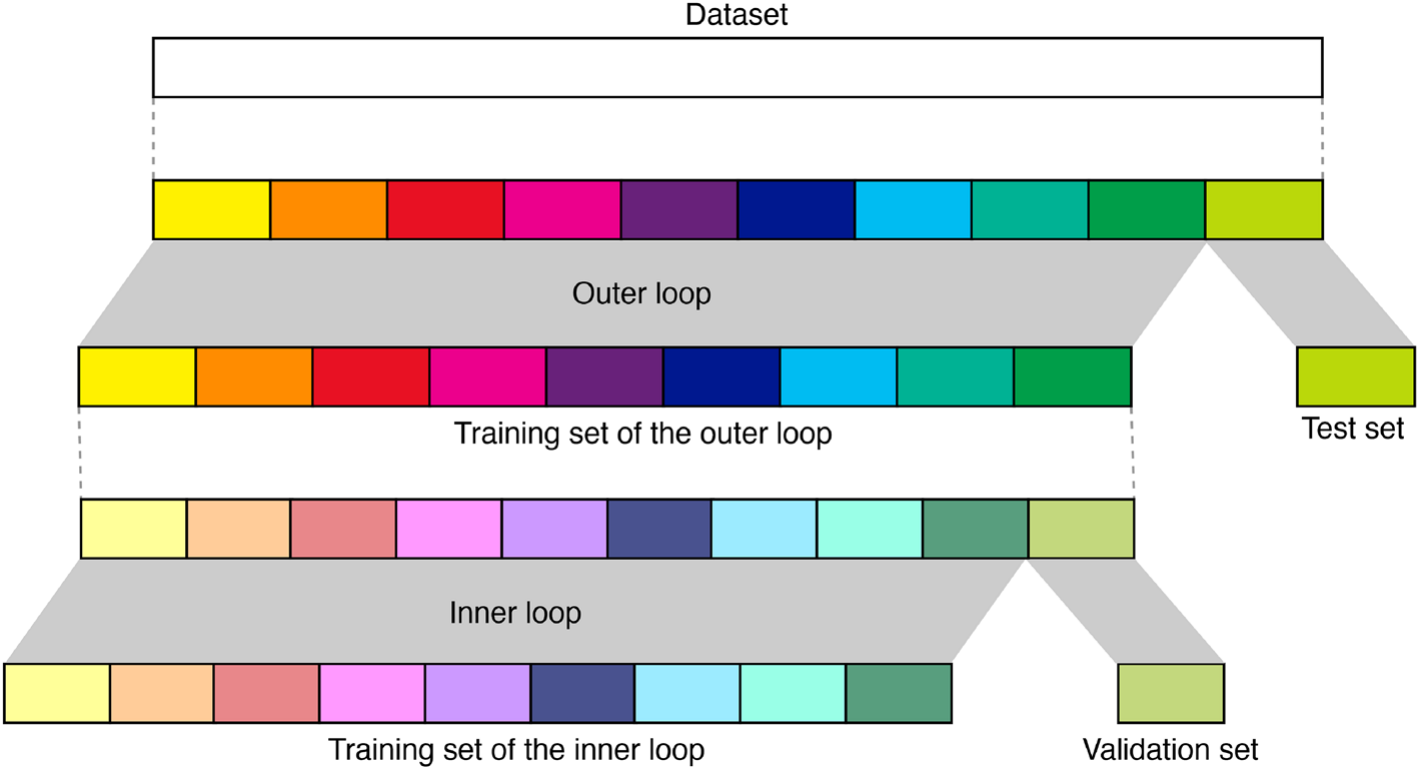
A scheme of tenfold nested CV is represented. The inner CV loop is used to perform feature selection, optimize hyper-parameters and select the best classifier, whereas the outer loop estimates the selected models’ performance.

To train, validate and test the classifier, we employed a tenfold nested stratified CV loop (Fig. 7). In particular, empirical evidence suggested that 5- or 10-fold CV should be preferred to leave-one-out (LOO) CV as consistently reported by both current literature^80–83^ and state-of-the-art machine learning development tools documentation (see, e.g., https://scikit-learn.org/stable/modules/cross_validation.html).

This strategy allowed us to perform, at the same time, in the inner CV loop, selection of the best feature combination in discriminating the presence of schizotypal traits, as well as the best classifier (between C-SVM with linear kernel and logistic regression with L2 penalty) along with their hyper-parameter optimization. Indeed, given that it is not possible to define a priori which is the best machine learning algorithm with respect to the data and the specific problem to address^84^, we used two well-established classifiers (C-SVM with linear kernel and logistic regression with L^2^ regularization), which are generally appropriate choices for reducing overfitting in a small sample. In particular, for a binary classification task, a C-SVM constructs a hyperplane in a high-dimensional space separating the training data into two classes. Since, in general, the larger the margin the lower the generalization error of the classifier, a good separation is achieved by the hyperplane that has the largest distance to the nearest training data points of any class^85^. On the other hand, logistic regression measures the relationship between the categorical dependent variable and one or more independent variables by estimating probabilities using a logistic sigmoid function^86^. The C hyperparameter of both the C-SVM and logistic regression classifiers takes a value that is proportional to the inverse of the regularization strength used during the training phase. For the C-SVM classifier, e.g., the choice of the C value is a trade-off between misclassification of training examples and simplicity of the decision surface. A low C value makes the decision surface smooth, while a high C value aims at classifying all training examples correctly. In this study, we varied the C value of both the C-SVM and logistic regression in the set {0.1, 0.2, 0.3}. We refer to specialized reference textbooks for a deeper description of these state-of-the-art systems^85–87^. Once the best estimator (determined by the best classifier/hyper-parameter/feature combination) maximizing the balanced accuracy was found in the inner CV, it was re-trained on the outer training set and tested on the test set kept out from the outer CV to obtain an unbiased estimation of the model’s prediction error. This procedure was repeated for each fold of the outer CV. Before each training (both in the inner and outer CV), each feature was standardized with reference to the training set only. Test set data were not used in any way during the learning process, thus preventing any form of peeking effect^88^.

Since the performance and the selected features may vary depending on how the data are split in each fold of the CV, we repeated the nested stratified CV procedure 1000 times recording the frequency that each feature combination was selected from each fold of the round of the outer CV. Average and standard deviation of the results from all repetitions in terms of sensitivity (the proportion of high schizotypes correctly identified as such), specificity (the proportion of low schizotypes correctly identified as such), balanced accuracy, and AUC were computed to get a final model assessment score in the test set of the outer-CV. The average ROC curve^77^ across 10 000 outer loops of the nested CV (10 folds × 1000 repetitions) along with the standard deviation and the 99.9% confidence interval of the average was also computed.

We used own code, freely available at https://github.com/sdiciotti/Schizotypy-prediction, developed in Python programming language (release 3.9.1) for data analysis using the scikit-learn module.

## References

[1] Klosterkötter J, Hellmich M, Steinmeyer EM, Schultze-Lutter F (2001). Diagnosing schizophrenia in the initial prodromal phase. Arch. Gen. Psychiatry.

[2] Kwapil TR, Gross GM, Silvia PJ, Barrantes-Vidal N (2013). Prediction of psychopathology and functional impairment by positive and negative schizotypy in the chapmans’ ten-year longitudinal study. J. Abnorm. Psychol..

[3] Barrantes-Vidal N, Grant P, Kwapil TR (2015). The role of schizotypy in the study of the etiology of schizophrenia spectrum disorders. Schizophr. Bull..

[4] Fenner B, Cooper N, Romei V, Hughes G (2020). Individual differences in sensory integration predict differences in time perception and individual levels of schizotypy. Conscious. Cogn..

[5] Ferri F, Venskus A, Fotia F, Cooke J, Romei V (2018). Higher proneness to multisensory illusions is driven by reduced temporal sensitivity in people with high schizotypal traits. Conscious. Cogn..

[6] Wu Y, Cao H, Baranova A (2020). Multi-trait analysis for genome-wide association study of five psychiatric disorders. Transl Psychiatry..

[7] Plomin R, Haworth CMA, Davis OSP (2009). Common disorders are quantitative traits. Nat. Rev. Genet..

[8] Ripke S (2013). Genome-wide association analysis identifies 13 new risk loci for schizophrenia. Nat. Genet..

[9] Bakan P, Peterson K (1994). Pregnancy and birth complications: a risk factor for schizotypy. J. Pers. Disord..

[10] Compton MT, Chien VH, Bollini AM (2009). Associations between past alcohol, cannabis, and cocaine use and current schizotypy among first-degree relatives of patients with schizophrenia and non-psychiatric controls. Psychiatr. Q..

[11] Yan X, Zhao X, Li J, He L, Xu M (2018). Effects of early-life malnutrition on neurodevelopment and neuropsychiatric disorders and the potential mechanisms. Prog. Neuro-Psychopharmacol. Biol. Psychiatry.

[12] Matheson SL, Shepherd AM, Laurens KR, Carr VJ (2011). A systematic meta-review grading the evidence for non-genetic risk factors and putative antecedents of schizophrenia. Schizophr. Res..

[13] Varese F (2012). Childhood adversities increase the risk of psychosis: a meta-analysis of patient-control, prospective-and cross-sectional cohort studies. Schizophr. Bull..

[14] De Sousa P, Varese F, Sellwood W, Bentall RP (2014). Parental communication and psychosis: a meta-analysis. Schizophr. Bull..

[15] Saha S, Scott JG, Varghese D, McGrath JJ (2013). Socio-economic disadvantage and delusional-like experiences: a nationwide population-based study. Eur. Psychiatry.

[16] Bourque F, Van Der Ven E, Malla A (2011). A meta-analysis of the risk for psychotic disorders among first- and second-generation immigrants. Psychol. Med..

[17] Lenzenweger MF (2006). Schizotypy an organizing framework for schizophrenia research. Curr. Dir. Psychol. Sci..

[18] Raine A (2006). Schizotypal personality: neurodevelopmental and psychosocial trajectories. Annu. Rev. Clin. Psychol..

[19] Debbané M, Mohr C (2015). Integration and development in schizotypy research: an introduction to the special supplement. Schizophr. Bull..

[20] Liu T (2019). Occipital alpha connectivity during resting-state electroencephalography in patients with ultra-high risk for psychosis and schizophrenia. Front. Psychiatry.

[21] Harris A, Melkonian D, Williams L, Gordon E (2006). Dynamic spectral analysis findings in first episode and chronic schizophrenia. Int. J. Neurosci..

[22] Clementz BA, Sponheim SR, Iacono WG, Beiser M (1994). Resting EEG in first-episode schizophrenia patients, bipolar psychosis patients, and their first-degree relatives. Psychophysiology.

[23] Goldstein MR, Peterson MJ, Sanguinetti JL, Tononi G, Ferrarelli F (2015). Topographic deficits in alpha-range resting EEG activity and steady state visual evoked responses in schizophrenia. Schizophr. Res..

[24] Hughes SW, Crunelli V (2005). Thalamic mechanisms of EEG alpha rhythms and their pathological implications. Neuroscientist.

[25] Schreckenberger M (2006). The thalamus as the generator and modulator of EEG alpha rhythm: a combined PET/EEG study with lorazepam challenge in humans. Neuroimage.

[26] Millett D (2001). Hans berger: from psychic energy to the EEG. Perspect. Biol. Med..

[27] Murphy M, Öngür D (2019). Decreased peak alpha frequency and impaired visual evoked potentials in first episode psychosis. NeuroImage Clin..

[28] Jin Y (2006). Therapeutic effects of individualized alpha frequency transcranial magnetic stimulation (αTMS) on the negative symptoms of schizophrenia. Schizophr. Bull..

[29] Friston KJ, Frith CD (1995). Schizophrenia: a disconnection syndrome?. Clin. Neurosci. (New York, N.Y.).

[30] Ito J, Nikolaev AR, Van Leeuwen C (2005). Spatial and temporal structure of phase synchronization of spontaneous alpha EEG activity. Biol. Cybern..

[31] Bahramisharif A (2013). Propagating neocortical gamma bursts are coordinated by traveling alpha waves. J. Neurosci..

[32] Halgren M (2019). The generation and propagation of the human alpha rhythm. Proc. Natl. Acad. Sci. U. S. A..

[33] Manjarrez E, Vázquez M, Flores A (2007). Computing the center of mass for traveling alpha waves in the human brain. Brain Res..

[34] Becker R, Van de Ville D, Kleinschmidt A (2018). Alpha oscillations reduce temporal long-range dependence in spontaneous human brain activity. J. Neurosci..

[35] Hanslmayr S, Gross J, Klimesch W, Shapiro KL (2011). The role of alpha oscillations in temporal attention. Brain Res. Rev..

[36] Samaha J, Bauer P, Cimaroli S, Postle BR (2015). Top-down control of the phase of alpha-band oscillations as a mechanism for temporal prediction. Proc. Natl. Acad. Sci. U. S. A..

[37] Koh Y (2011). An MEG study of alpha modulation in patients with schizophrenia and in subjects at high risk of developing psychosis. Schizophr. Res..

[38] Fuggetta G, Bennett MA, Duke PA, Young AMJ (2014). Quantitative electroencephalography as a biomarker for proneness toward developing psychosis. Schizophr. Res..

[39] Tandon N, Tandon R (2019). Using machine learning to explain the heterogeneity of schizophrenia. Realizing the promise and avoiding the hype. Schizophr. Res..

[40] Noor MBT, Zenia NZ, Kaiser MS, Mamun SA, Mahmud M (2020). Application of deep learning in detecting neurological disorders from magnetic resonance images: a survey on the detection of Alzheimer’s disease, Parkinson’s disease and schizophrenia. Brain Inform..

[41] Zhang, L. EEG Signals Classification Using Machine Learning for the Identification and Diagnosis of Schizophrenia. *Proc. Annu. Int. Conf. IEEE Eng. Med. Biol. Soc. EMBS* 4521–4524 (2019). doi:10.1109/EMBC.2019.8857946 10.1109/EMBC.2019.8857946 31946870

[42] Johannesen JK, Bi J, Jiang R, Kenney JG, Chen C-MA (2016). Machine learning identification of EEG features predicting working memory performance in schizophrenia and healthy adults. Neuropsychiatric Electrophysiol..

[43] Thilakavathi B, Shenbaga Devi S, Malaiappan M, Bhanu K (2019). EEG power spectrum analysis for schizophrenia during mental activity. Australas. Phys. Eng. Sci. Med..

[44] Iwabuchi SJ, Palaniyappan L (2017). Abnormalities in the effective connectivity of visuothalamic circuitry in schizophrenia. Psychol. Med..

[45] Raine A (1991). The SPQ: a scale for the assessment of schizotypal personality based on DSM-III-r criteria. Schizophr. Bull..

[46] St. Louis, E. *et al. Electroencephalography (EEG): An Introductory Text and Atlas of Normal and Abnormal Findings in Adults, Children, and Infants*. *Electroencephalography (EEG): An Introductory Text and Atlas of Normal and Abnormal Findings in Adults, Children, and Infants* (2016). doi:10.5698/978-0-9979756-0-4 27748095

[47] Narayanan B (2014). Resting state electroencephalogram oscillatory abnormalities in schizophrenia and psychotic bipolar patients and their relatives from the bipolar and schizophrenia network on intermediate phenotypes study. Biol. Psychiatry.

[48] Garrity AG (2007). Aberrant ‘default mode’ functional connectivity in schizophrenia. Am. J. Psychiatry.

[49] Öngür D (2010). Default mode network abnormalities in bipolar disorder and schizophrenia. Psychiatry Res. Neuroimaging.

[50] Omori M (1995). Quantitative EEG in never-treated schizophrenic patients. Biol. Psychiatry.

[51] Jetha MK, Schmidt LA, Goldberg JO (2009). Resting frontal eeg asymmetry and shyness and sociability in schizophrenia: a pilot study of community-based outpatients. Int. J. Neurosci..

[52] Merrin EL, Floyd TC (1992). Negative symptoms and EEG alpha activity in schizophrenic patients. Schizophr. Res..

[53] Yozawitz A (1979). Dichotic perception: evidence for right hemisphere dysfunction in affective psychosis. Br. J. Psychiatry.

[54] Walker E, McGuire M (1982). Intra- and interhemispheric information processing in schizophrenia. Psychol. Bull..

[55] Gur RE (1978). Left hemisphere dysfunction and left hemisphere overactivation in schizophrenia. J. Abnorm. Psychol..

[56] Fan X, Miller BC, Park K, Christensen M, Tai RH (2015). An exploratory study about inaccuracy and invalidity in adolescent self-report surveys. Field Methods.

[57] Austin EJ, Deary IJ, Gibson GJ, Mcgregort MJ, Dent JB (1998). Individual response spread in self-report scales: personality correlations and consequences. Pers. Individ. Differ..

[58] Doesburg SM, Green JJ, McDonald JJ, Ward LM (2009). From local inhibition to long-range integration: a functional dissociation of alpha-band synchronization across cortical scales in visuospatial attention. Brain Res..

[59] Cecere R, Rees G, Romei V (2015). Individual differences in alpha frequency drive crossmodal illusory perception. Curr. Biol..

[60] Samaha J, Postle BR (2015). The speed of alpha-band oscillations predicts the temporal resolution of visual perception. Curr. Biol..

[61] Minami S, Amano K (2017). Illusory jitter perceived at the frequency of alpha oscillations. Curr. Biol..

[62] Migliorati D (2020). Individual alpha frequency predicts perceived visuotactile simultaneity. J. Cogn. Neurosci..

[63] Cooke J, Poch C, Gillmeister H, Costantini M, Romei X (2019). Oscillatory properties of functional connections between sensory areas mediate cross-modal illusory perception. J. Neurosci..

[64] Wutz A, Melcher D, Samaha J (2018). Frequency modulation of neural oscillations according to visual task demands. Proc. Natl. Acad. Sci. U. S. A..

[65] Battaglini L (2020). The effect of alpha tACS on the temporal resolution of visual perception. Front. Psychol..

[66] Ronconi L, Busch NA, Melcher D (2018). Alpha-band sensory entrainment alters the duration of temporal windows in visual perception. Sci. Rep..

[67] World Medical Association (2001). World Medical Association Declaration of Helsinki: ethical principles for medical research involving human subjects. Bull. World Health Organ..

[68] Barry RJ, Clarke AR, Johnstone SJ, Magee CA, Rushby JA (2007). EEG differences between eyes-closed and eyes-open resting conditions. Clin. Neurophysiol..

[69] Delorme A, Makeig S (2004). EEGLAB: An open source toolbox for analysis of single-trial EEG dynamics including independent component analysis. J. Neurosci. Methods.

[70] Bell AJ, Sejnowski TJ (1995). An information-maximization approach to blind separation and blind deconvolution. Neural Comput..

[71] Onton J, Delorme A, Makeig S (2005). Frontal midline EEG dynamics during working memory. Neuroimage.

[72] Makeig S (2004). Electroencephalographic brain dynamics following manually responded visual targets. PLoS Biol..

[73] Vinck M, Oostenveld R, Van Wingerden M, Battaglia F, Pennartz CMA (2011). An improved index of phase-synchronization for electrophysiological data in the presence of volume-conduction, noise and sample-size bias. Neuroimage.

[74] Campos Viola F (2009). Semi-automatic identification of independent components representing EEG artifact. Clin. Neurophysiol..

[75] Di Gregorio F, Ernst B, Steinhauser M (2019). Differential effects of instructed and objective feedback reliability on feedback-related brain activity. Psychophysiology.

[76] Corcoran AW, Alday PM, Schlesewsky M, Bornkessel-Schlesewsky I (2018). Toward a reliable, automated method of individual alpha frequency (IAF) quantification. Psychophysiology.

[77] Lee H (2019). Relationship of critical dynamics, functional connectivity, and states of consciousness in large-scale human brain networks. Neuroimage.

[78] Cohen MX (2014). Analyzing Neural Time Series Data: Theory and Practice.

[79] Šidák Z (1967). Rectangular confidence regions for the means of multivariate normal distributions. J. Am. Stat. Assoc..

[80] Breiman L, Spector P (1992). Submodel selection and evaluation in regression. The X-random case. Int. Stat. Rev. Rev. Int. Stat..

[81] Hastie T, Tibshirani R, Friedman J (2009). The Elements of Statistical Learning. The Business of Giving.

[82] James G, Witten D, Hastie T, Tibshirani R (2013). An Introduction to Statistical Learning Current Medicinal Chemistry.

[83] Kohavi, R. A Study of Cross-Validation and Bootstrap for Accuracy Estimation and Model Selection. in *Proceedings of the 14th International Joint Conference on Artificial Intelligence - Volume 2* 1137–1143 (Morgan Kaufmann Publishers Inc., 1995).

[84] Wolpert, D. H. *The Lack of a Priori Distinctions between Learning Algorithms*. *Neural Computation* **8**, (1996).

[85] Bishop, C. M. Sparse Kernel Machines. in *Pattern Recognition and Machine Learning, Information Science and Statistics* (Springer, New York, 2006).

[86] Bishop, C. M. Logistic Regression. in *Pattern Recognition and Machine Learning, Information Science and Statistics* (Springer, New York, 2006). doi:10.1108/k.2010.06739hae.001

[87] Alpaydin E (2010). Introduction to Machine Learning. Adaptive Computation and Machine Learining.

[88] Diciotti, S., Ciulli, S., Mascalchi, M., Giannelli, M., Toschi, N. The “ Peeking ” Effect in Supervised Feature Selection on Diffusion Tensor Imaging Data. **34**, 3685 (2013). 10.3174/ajnr.A3685 PMC7965616 23868167

