# Publisher Correction: Resting state alpha oscillatory activity is a valid and reliable marker of schizotypy

**DOI:** 10.1038/s41598-021-92571-8

**Published:** 2021-06-23

**Authors:** Jelena Trajkovic, Francesco Di Gregorio, Francesca Ferri, Chiara Marzi, Stefano Diciotti, Vincenzo Romei

**Affiliations:** 1 Centro Studi e Ricerche in Neuroscienze Cognitive, Dipartimento di Psicologia, Alma Mater Studiorum – Università di Bologna, Campus di Cesena, 47521 Cesena, Italy; 2 UO Medicina Riabilitativa e Neuroriabilitazione, Azienda Unità Sanitaria Locale, 40139 Bologna, Italy; 3 Department of Neuroscience, Imaging and Clinical Sciences, “G. d’Annunzio” University of Chieti-Pescara, Chieti, Italy; 4 Institute for Advanced Biomedical Technologies, “G. d’Annunzio” University of Chieti-Pescara, Chieti, Italy; 5 Department of Electrical, Electronic, and Information Engineering “Guglielmo Marconi”, University of Bologna, Cesena, Italy; 6 Alma Mater Research Institute for Human‑Centered Artificial Intelligence, University of Bologna, Bologna, Italy; 7 IRCCS Fondazione Santa Lucia, 00179 Rome, Italy

Correction to: *Scientific Reports* https://doi.org/10.1038/s41598-021-89690-7, published online 17 May 2021

The original version of this Article contained an error in Reference 6, which was incorrectly given as:

Wu, Y. *et al.* Multi-trait analysis for genome-wide association study of five psychiatric disorders. *Transl. Psychiatry* https://doi.org/10.1038/s41398-020-00924-0 (2020).

The correct reference is listed below:

Wu, Y., Cao, H., Baranova, A. *et al.* Multi-trait analysis for genome-wide association study of five psychiatric disorders. *Transl Psychiatry* **10,** 209 (2020). https://doi.org/10.1038/s41398-020-00902-6

The original Article has been corrected.

